# Are we doing our best to contain the spread of West Nile virus? Evaluating intervention efficacy through mathematical modelling

**DOI:** 10.1186/s13071-025-07128-9

**Published:** 2025-12-05

**Authors:** Elisa Fesce, Giovanni Marini, Roberto Rosà, Davide Lelli, Monica Pierangela Cerioli, Mario Chiari, Marco Farioli, Nicola Ferrari

**Affiliations:** 1 Department of Veterinary Medicine and Animal Sciences, Wildlife Health Laboratory, Università degli Studi di Milano, Lodi, LO Italy; 2 Research and Innovation Centre, Fondazione Edmund Mach, San Michele all’Adige, TN Italy; 3 Centre Agriculture Food Environment, University of Trento, San Michele all’Adige, TN Italy; 4 Istituto Zooprofilattico Sperimentale della Lombardia e dell’emilia Romagna “B. Ubertini”, Brescia, Italy; 5 Regione Lombardia UO Veterinaria Direzione Generale Welfare, Piazza Città di Lombardia, 1, Milano, Italy; 6 Regione Lombardia, ATS Insubria, UO Veterinaria DG Welfare, 21100 Varese, Italy

**Keywords:** Vector-borne infections, Mosquito control, Disease control, Intervention strategies, Epidemiology, Infectious disease dynamics, Infection transmission, Public health, Ecological modelling

## Abstract

**Background:**

West Nile virus (WNV) is an emerging vector-borne pathogen that is becoming increasingly prevalent in temperate regions. The development of effective intervention strategies is crucial for limiting its spread; however, the adaptability and ubiquity of mosquitoes, combined with the complexity of the WNV transmission cycle, continue to hinder its eradication.

**Methods:**

This study employs a deterministic compartmental model to evaluate the effectiveness of ten intervention strategies targeting either the mosquito (vector) or avian (host) population in the Lombardy region of Italy.

**Results:**

Vector-targeted interventions were more effective than host-targeted measures, with breeding site reduction and larvicide treatments demonstrating the greatest efficacy. In contrast, interventions targeting adult mosquitoes, including adulticide treatments and elimination of overwintering mosquitoes, showed moderate efficacy. Furthermore, the impact of eliminating overwintering mosquitoes gradually diminished over time. Host-targeted strategies, such as bird population reduction, were ineffective and, in some cases, led to increased WNV transmission. The efficacy of all interventions varied temporally, peaking in mid-summer.

**Conclusions:**

These findings highlight the importance of prioritising mosquito control, particularly targeting immature stages, to mitigate WNV outbreaks. Our study highlights the critical role of mathematical modelling in designing effective intervention strategies. By providing a structured framework to evaluate and predict the outcomes of various approaches, modelling can aid disease control while optimising resource allocation and minimising environmental impact. Mathematical models, therefore, prove to be powerful tools for balancing public health goals with sustainable practices.

**Graphical Abstract:**

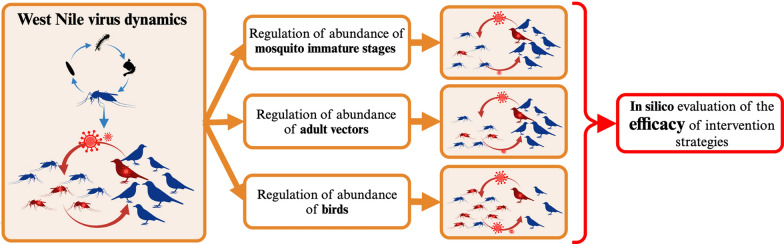

**Supplementary Information:**

The online version contains supplementary material available at 10.1186/s13071-025-07128-9.

## Background

Vector-borne diseases are estimated to represent 17% of all infectious diseases, causing more than 700,000 deaths worldwide every year [1, 2] and overwhelming the health system in many countries. Vector control plays a key role in controlling vector-borne infections [2], but the distribution of vectors and the epidemiological features of the infections they transmit are often determined by a complex set of demographic, environmental and social factors.

In the context of public health, mosquitoes are among the most significant vectors of infection. These insects can transmit a wide range of diseases, including malaria, chikungunya, dengue, Zika and West Nile [3, 4]. The latter is transmitted by a flavivirus [West Nile virus (WNV)] and has raised concerns in recent years in several temperate countries, given its increasing prevalence and the potential for serious public health implications. WNV is maintained in an enzootic cycle involving mosquitoes, mainly those of the *Culex* genus, and birds but can infect humans and other mammals if bitten by an infectious mosquito. Several mosquito species within and beyond the *Culex* genus have been found competent for WNV transmission [1, 5–12], and more than 300 avian species have been identified as positive for WNV infection [13], indicating that diverse vectors and host communities may contribute variably to WNV dynamics.

In humans, most infections are asymptomatic, with approximately 20% of cases resulting in symptoms, which typically present as a mild flu-like syndrome. However, approximately 1 in 150 individuals may develop severe disease, including neurological complications, which can sometimes lead to death [14, 15]. Nevertheless, the spread and emergence of infections in new regions, the rising number of human cases in endemic areas and the lack of vaccines for humans are significant public health concerns, underscoring the urgent need for effective surveillance and intervention strategies [16–18].

The efficacy of an intervention strategy is contingent upon several factors, including the targeted developmental stages of mosquitoes (eggs, larvae, pupae and adults), the timing of application and the intensity of the treatment. For instance, in 2015, approximately US$2.9 billion was allocated globally to malaria control, highlighting the substantial financial investment required for vector control efforts [2]. While the implementation of vector control measures has been demonstrated to be among the most cost-effective public health interventions, insufficient levels of funding and poor programme implementation have frequently resulted in a considerable burden of vector-borne diseases in numerous countries [2]. A range of vector control methods have been proposed, including larval control, adult mosquito control, personal protection measures and integrated vector management combining these strategies. However, the effectiveness of these methods is frequently constrained by limitations such as environmental risks, short-term impacts on adult mosquito populations and difficulties in achieving public compliance [16]. As emphasised by Bellini and colleagues, further research, particularly in Europe, is necessary to assess the efficacy of these methods, optimise their spatial and temporal application and improve decision-making processes for the prevention and control of WNV outbreaks [16]. In addition to mosquito-focused interventions, certain bird species that serve as reservoir hosts for WNV and are considered for surveillance could be targeted for control measures to mitigate viral spread. Even if not directly for viral control, invasive bird species may be managed, and the impact of such control efforts on viral transmission remains an area of significant interest. This situation is further complicated by the fact that bird species can have different competences for WNV transmission, with some species being competent and others not [19]. Furthermore, even among competent species, competence is variable [19], potentially affecting the efficacy of controlling bird populations.

Mathematical modelling plays a crucial role in investigating the mechanisms driving WNV infection [17, 20–25]. In regions such as Veneto, Emilia-Romagna and Lombardy (northern Italy), data from entomological surveillance plans for arboviruses have been successfully utilised to study WNV spread and its maintenance mechanisms [25–27]. This approach provides a dynamic evaluation of the system, analysing its changes over time, and can simulate potential scenarios, such as the application of different intervention strategies, before field testing, minimising time and resource consumption. We therefore propose a theoretical framework, based on a system of differential equations, that simulates intervention strategies to limit WNV circulation among resident mosquitoes. Since reducing either the number of hosts or vectors might help control infections, we simulated intervention strategies targeting avian hosts as well as different developmental stages of mosquitoes (eggs, larvae, pupae or adults). Each intervention was tested for different removal rates to account for potential differences in strategy efficacy. Given the specificity of WNV dynamics to local climatic and environmental conditions and effects of modelling choices on model control implications [28], our model is based on a computational framework that uses mathematical models calibrated through a Bayesian approach to simulate mosquito population dynamics and WNV infection rates. The framework, described in detail in the dedicated section below (“Model framework”), has been previously validated for different Italian regions [25–27] and was fitted to data on mosquito abundance and WNV prevalence in the vector population collected in the Lombardy region [27].

## Methods

### Study area and dataset

Since its first detection in the Tuscany region in Italy in 1998 [29], seasonal viral circulation has been confirmed yearly in wild reservoirs and humans in several regions (including Lombardy, Veneto and Emilia-Romagna), with unexpectedly high numbers of human infections in 2018 and 2022 [25, 30, 31]. This study relies on previously collected entomological data to develop and validate a model of WNV dynamics in the Lombardy region, building on an already validated mathematical modelling framework [25–28]. Intervention strategies, in contrast, were assessed in a theoretical context by exploring variations in model outcomes under different assumptions.

**Fig. 1:**
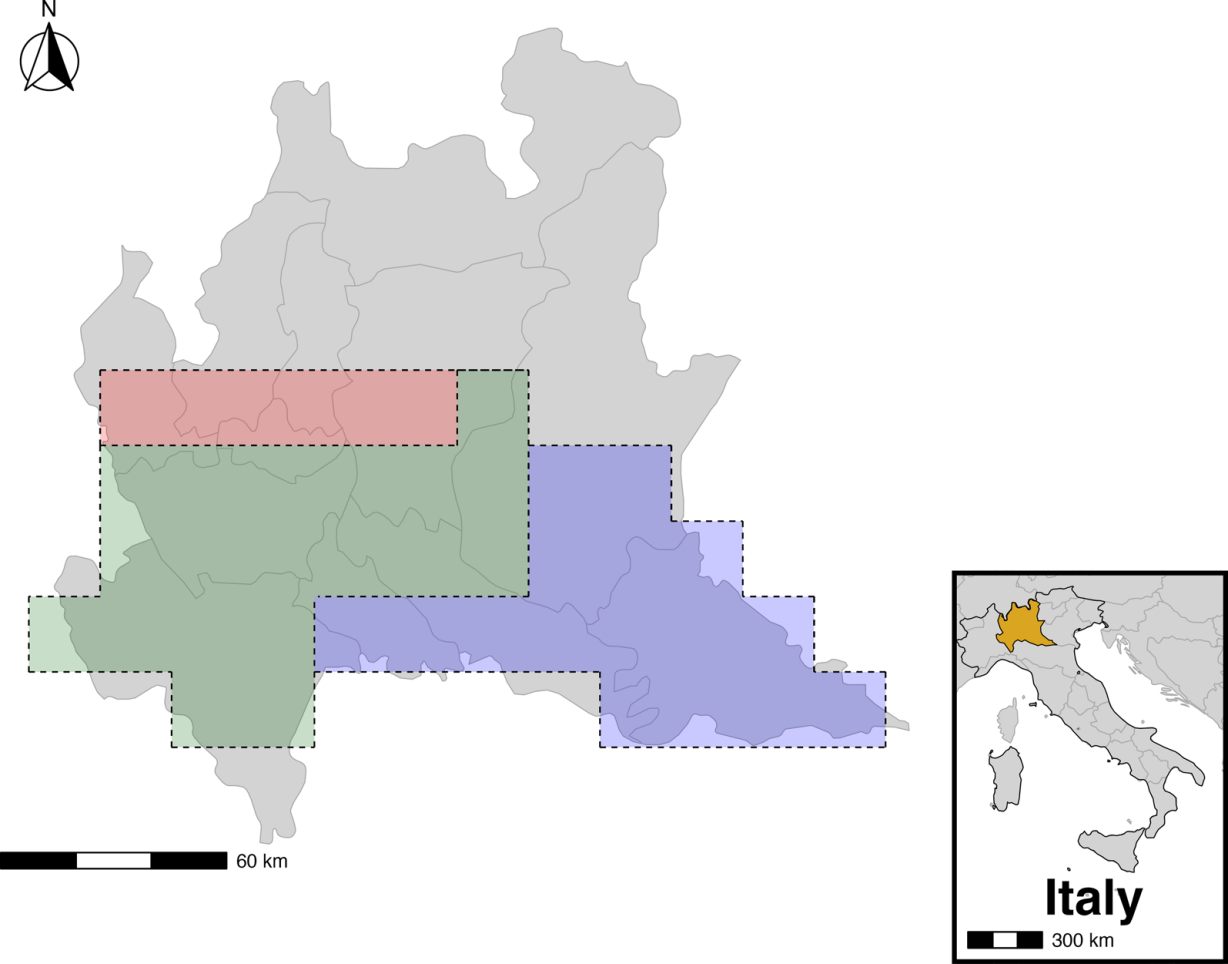
Schematic representation of the study area. The red area represents the northern subregion, the green area represents the western subregion and the blue area represents the eastern subregion of the Lombardy region (Italy). The image was created using the R package *rnaturalearth*

Mosquitoes were collected every 15 days by Regione Lombardia and Istituto Zooprofilattico Sperimentale della Lombardia ad Emilia-Romagna in the Lombardy region between April and October in 2016, 2017 and 2018 as part of the national surveillance programme for arboviruses using CO_2_ traps [Centers for Disease Control and Prevention (CDC)-CO_2_ traps]. Each trap covered an area of at most 400 km^2^ [32]. Mosquito samples were then pooled according to their species (up to 100 mosquitoes per pool), and all *Culex pipiens* (*Cx. pipiens)* pools were tested for WNV presence via polymerase chain reaction (PCR). Following previous analyses [27], to account for intraregional differences in temperature, precipitation, mosquito abundance and WNV presence, the study area was divided into three different subregions (northern, eastern and western, as shown in Fig. 1). All analyses were conducted for only the western and eastern subregions, as WNV was not detected in the northern subregion during the study period.

### Model framework

#### Base model

Deterministic compartmental models were used to simulate the number of infected mosquitoes and birds. These models were then used to produce ten scenarios in which different intervention strategies were applied. The computational framework used to estimate WNV dynamics in the Lombardy region, summarised in Additional file 1: Figs. S1 and S2 (*base model*), follows the one proposed in Fesce et al. [27], where WNV dynamics are described through a system of 11 differential equations representing transmission between competent birds (here considered *Pica pica*, the Eurasian magpie) and *Cx. pipiens* mosquitoes. We modified the model to include a single non-competent avian species (or community of non-competent species) in the WNV cycle and to fit it with the abundance of mosquitoes (*Cx. pipiens*) and their WNV prevalence recorded in our study area. Due to the general scarcity of information regarding the bird compartment – in terms of both absolute and relative abundances as well as parameterisation – we included only one competent and one non-competent bird species. These can be interpreted either as a single competent species (*Pica pica*, the Eurasian magpie) and one non-competent species or as aggregated compartments representing all competent and all non-competent species, respectively.

Specifically, the framework consists of a combination of two compartmental models, a first model to estimate the daily number of *Cx. pipiens* female mosquitoes in the study area (entomological model) and a second model to simulate the dynamics of WNV infection between birds and mosquitoes (epidemiological model). Both models were fitted to data gathered by the surveillance of ongoing arboviruses in the Lombardy region [27, 32]. The entomological model is a deterministic model based on a system of four differential equations representing the developmental stages of mosquitoes (eggs, larvae, pupae and adult females. The full set of equations is reported in Additional file 1: Text S1). The second model (epidemiological model) incorporates the number of female *Cx. pipiens* mosquitoes estimated by the first model and simulates WNV transmission between mosquitoes and avian host populations. It is based on a system of 11 differential equations for estimating WNV circulation in the study area (the full set of equations is reported in Additional file 1: Text S2). Briefly, the modelling framework simulates WNV infection dynamics between competent birds (Eurasian magpies) and mosquitoes (*Cx. pipiens*), using published estimates of adult female mosquito abundance [27]. Transmission occurs via mosquito bites, with rates depending on the number of infectious birds and mosquitoes, biting frequency and the proportion of bites on birds. Birds follow a susceptible–exposed–infected–recovered (SEIR) model, recovering with lifelong immunity, whereas mosquitoes follow a susceptible–exposed–infected (SEI) model, remaining infectious for life because of their short lifespan. The incubation periods for both hosts are included.

In line with previous models [25–27], birds enter the system as susceptible according to their natural birth rate and die because of natural mortality. In accordance with laboratory and field experiments, no mortality due to infection was included in the model [33, 34]. The parameter values for the model simulation were estimated from the literature following Marini et al. [25]. Given the importance of temperature for the developmental rates of *Cx. pipiens* and for WNV transmission rates [35–38], the models incorporate the following parameters as functions of the mean daily temperature: developmental and death rates for eggs, larvae and pupae; gonotrophic cycle length; the death rate for eggs; the death rate for larvae; adult longevity (in the entomological model); the probability of WNV transmission from birds to mosquitoes per infectious bite; and extrinsic incubation (in the epidemiological model). The temperature dependence of the parameters is based on the formulas and estimates reported in Marini et al. [25]. We considered the mean daily temperature across the 3 years of investigation and different locations from meteorological records collected by *ARPA Lombardia*. See Additional file 1: Table S1 and S2 for the full list of parameter values.

In line with the reference models [25] and owing to the limited availability of data on the epidemiological parameters of European bird species, as well as on the composition and abundance of bird communities in the areas surrounding mosquito trapping sites, the following parameters were treated as unknown: the initial number of birds in the area, the proportion of competent bird species, the proportion of mosquito bites on birds, the initial number of immune birds at the start of the season (April) and the avian recovery rate (Table S3, Appendix 1). Similarly, due to the lack of available data, the prevalence of infectious mosquitoes at the beginning of the season (April) was also considered unknown. Unknown free parameter distributions for both models were estimated using a Bayesian Markov chain Monte Carlo (MCMC) approach, fitting the number of *Cx. pipiens* female mosquitoes and the estimated WNV mosquito prevalence between 2016 and 2018 [32], according to the method proposed in Marini et al. [25–27]. To estimate the free parameters, 10,000 iterations of MCMC were performed, and to avoid autocorrelation among estimates, 100 sets of parameters were chosen, resulting in 100 simulations accounting for parameter estimate variability. Convergence of the MCMC was checked by visual inspection of the posterior distribution of the parameters. The full list of parameter prior distributions (Table S3) and their posterior distributions estimated through MCMC (Fig. S3) are reported in Additional file 1. The model fit was visually assessed, and the number of observations falling within the 95% confidence intervals of the predictions was estimated (Additional file 1, Table S3 and Fig. S4).

#### Intervention models

To assess and compare the efficacy of different intervention strategies, we incorporated ten intervention strategies (Roman numerals from i to x), one at a time, in the base model as follows:

i. Use of an adulticide targeting overwintering adult mosquitoes (hereafter, elimination of overwintering mosquitoes): The reduction in the number of *Cx. pipiens* mosquitoes that survived the winter was simulated by reducing the number of mosquitoes at the beginning of each simulated year.
ii. Use of an adulticide during the mosquito activity period (adulticide treatment): To simulate an insecticide treatment, we included an additional death rate for *Cx. pipiens* adult mosquitoes in the entomological model. We considered this intervention strategy to be performed once every 2 weeks from the beginning to the end of the mosquito activity period, and it immediately killed a given fraction of the adult population.
iii. Use of a larvicide during the mosquito activity period (larvicide treatment): To simulate a larvicidal treatment, we included an additional death rate for *Cx. pipiens* eggs and larvae in the entomological model. The intervention strategy was considered to have a daily constant effect during the entire mosquito activity period by assuming that the effect of one treatment lasted until the following treatment, without any efficacy loss.
iv. Reduction of the number of breeding sites (mosquito breeding site reduction): To simulate the reduction in mosquito breeding sites (all possible sources of stagnant water), we reduced the density-dependent scaling factor driving the carrying capacity for the larval stages of the mosquito population in the entomological model, thus obtaining new mosquito abundances to include in the epidemiological model to simulate WNV spread. This intervention strategy was implemented continuously throughout the mosquito activity period.
v. Active removal of both competent and non-competent birds (removal of birds): To simulate the active removal of birds (both competent and non-competent), we included an additional death rate for both adult and young birds of both competent and non-competent populations of birds in the epidemiological model. We considered this intervention strategy to be performed once every month from the beginning to the end of the mosquito activity period, immediately culling a given fraction of the adult bird population.
vi. Active removal of competent birds (removal of competent birds): Analogous to treatment *iv*, to simulate the active removal of competent birds, we included an additional death rate in the epidemiological model for both adult and young birds of the competent population only. This intervention was performed once every month from the beginning to the end of the mosquito activity period.
vii. Active removal of non-competent birds (removal of non-competent birds): Analogous to treatments v and vi, we included an additional death rate for both adult and young birds of the non-competent population in the epidemiological model. This intervention was performed once every month from the beginning to the end of the mosquito activity period.
viii. Reduction in the number of breeding sites for the avian population (reduction in bird breeding sites): To simulate the reduction in breeding sites for the avian population, we reduced the environmental carrying capacity of both competent and non-competent bird populations in the epidemiological model. This intervention strategy was considered continuous during the mosquito activity period.
ix. Reduction of the number of breeding sites for competent birds (competent bird breeding site reduction): To simulate the reduction in breeding sites for the competent population, we reduced the environmental carrying capacity of competent birds in the epidemiological model. This intervention strategy was considered continuous during the mosquito activity period.
x. Reduction in the number of breeding sites for non-competent birds (non-competent bird breeding site reduction): To simulate the reduction in breeding sites for the non-competent population, we reduced the environmental carrying capacity of non-competent birds in the epidemiological model. This intervention strategy was considered continuous during the mosquito activity period.

As intervention strategies in the field are partly shaped by national regulations but also influenced by decisions made at the municipal level and by private citizens regarding their own properties, we selected plausible ranges for intervention durations based on expert opinion and modelling assumptions. In accordance with these durations, detailed in the description of each strategy, the intervention was incorporated into the model by assigning a non-zero value to the corresponding parameter during the active period and zero otherwise.

**Fig. 2:**
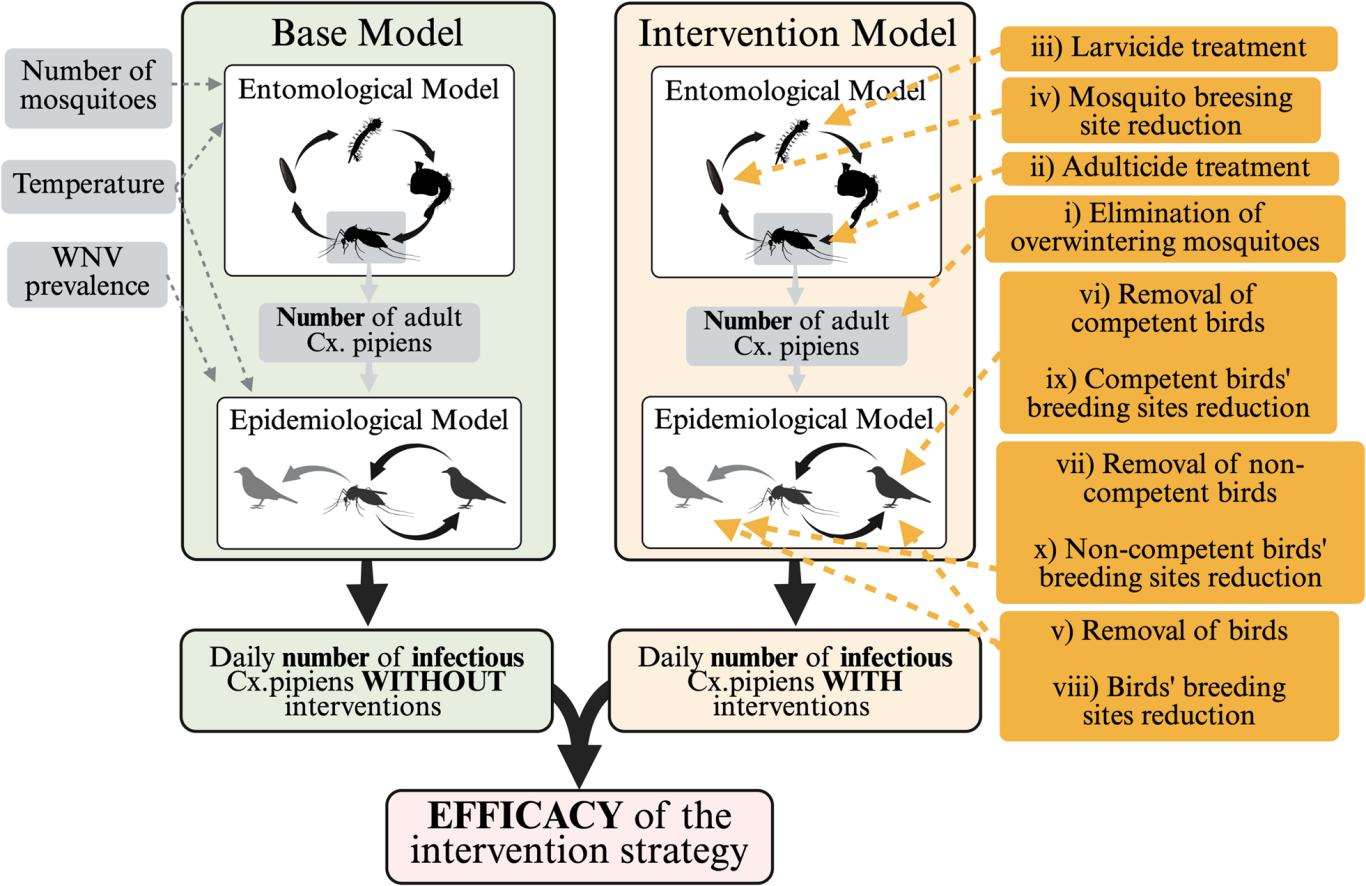
Schematic representation of the base model and the intervention model. White boxes represent the computational frameworks, while grey and orange boxes indicate the model inputs and simulated intervention strategies, respectively. The green and orange pathways depict the workflows of the base and intervention models, respectively, culminating in the estimation of intervention strategy efficacy (dark orange boxes). Dark orange circles in the intervention model workflow highlight the target species for the intervention strategy. Created in BioRender. Fesce, E. (2025) https://BioRender.com/gzxsr3u

We therefore theoretically evaluated the ability of these intervention strategies to reduce the daily number of infectious mosquitoes from the end of April to the end of October 2016–2018. For each intervention strategy, three different intensity levels were simulated (daily intensity rates of 0.2, 0.5 and 0.8). The process is schematically represented in Fig. 2, and the full set of equations used is reported in Additional file 1: Text S4.

For the inclusion of intervention strategies and the estimation of their efficacy, we considered a general scenario in which interventions are implemented theoretically, assuming their efficacy depends solely on the intrinsic characteristics of the intervention itself, independent of the ecological context in which they are applied.

#### Efficacy of the interventions

We defined the efficacy of an intervention (*E*_i_) as its capacity to reduce the mean daily number of infectious mosquitoes with respect to the base condition in the absence of any interventions. Furthermore, this method allows comparisons between interventions on the vector population and interventions on the host population.

Given the model structure, which simulates 100 scenarios of WNV dynamics for each year and area based on 100 different parameter sets, we calculated the daily mean number of infected mosquitoes in each subregion and year for the base model (*M*_*I*_) and the intervention model (*M*_I,i_). We then estimated the efficacy of each intervention for each simulation and averaged these estimates per year and subregion. To account for potential interannual and spatial variation in intervention efficacy, we calculated the mean efficacy across simulations and reported the overall mean across all years and areas, along with its 95% confidence intervals. The efficacy of the interventions was estimated as follows:

$$E_{i} \left( t \right) = \frac{{M_{I} \left( t \right) - \mathop {M_{I,i} \left( t \right)}\limits^{`} }}{{M_{I} \left( t \right)}}$$

where $\mathop {M_{I,i} \left( t \right)}\limits^{`}$ represents the mean number of infected mosquitoes on day *t* estimated by the *intervention model,* including the i-th intervention strategy, and *M*_*I*_(*t*) represents the daily number of infected mosquitoes estimated by the *base model*. Consequently, any intervention not resulting in a reduction in the number of infectious mosquitoes has $E_{i} = 0$; while $E_{i} = 1$ ($100{\text{\% }}$ from here on) corresponds to the elimination of all infectious mosquitoes. Any interventions resulting in $E_{i} < 0$ imply an increase in the number of infectious mosquitoes. While alternative approaches based on statistical comparisons between output distributions could be considered, we chose to compare outcomes from the same parameter sets before and after the intervention to minimise the influence of parameter uncertainty and ensure a conservative and robust evaluation of intervention efficacy.

## Results

### Efficacy of intervention strategies over time

**Fig. 3:**
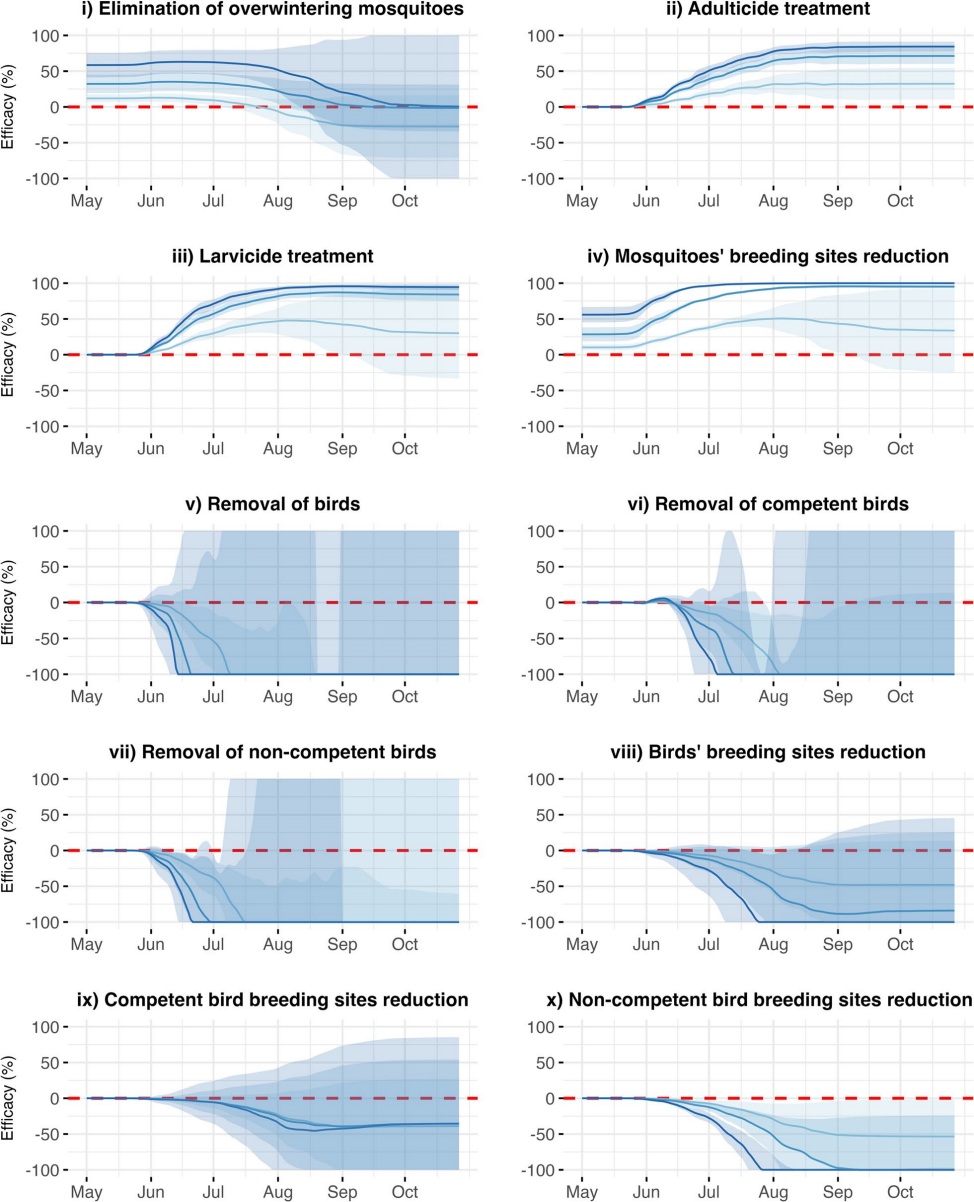
Efficacy of the intervention strategies over time. Each panel illustrates the efficacy (*E*_i_, *y*-axis) of a specific intervention strategy in reducing the number of infectious mosquitoes over time (*x*-axis). The solid lines represent the mean efficacy of interventions across years and areas, while the shaded areas represent the 95% quantiles calculated across years and areas. Different intensity rates are depicted in light blue, blue and dark blue, corresponding to rates of 0.2, 0.5 and 0.8, respectively. The dashed red line indicates the threshold (*E*_i_ = 0) separating effective (*E*_i_ > 0) from ineffective (*E*_i_ < 0) interventions. Panels (i) to (x) display the results for the corresponding intervention strategy

All tested interventions affected the number of infectious mosquitoes from April to October, with effects that varied over time. In all panels of Fig. 3, the solid lines, representing the mean efficacy of the intervention strategies averaged over the 3 years and two study areas, are never equal to zero (dashed red lines), indicating that the interventions consistently had some effect. Detailed results on efficacy across the 3 years and two subareas are presented in Fig. S5 of Additional file 1, together with a comprehensive analysis of the predicted number of infectious mosquitoes, shown in Figs. S6, S7 and S8. In detail, the reduction in mosquito breeding sites, which can be interpreted as all possible sources of standing water (Fig. 3iv), demonstrated the highest overall efficacy, as expected, exceeding 99% with an intervention intensity rate of 0.8 and over 90% at a rate of 0.5. The simulations emphasise that the efficacy of the intervention strategy remained consistent throughout the season. In addition to its high efficacy, the intervention showed consistent performance across years and areas (i.e. a consistent trend across all three simulation years and in both study areas, indicating that the intervention’s performance did not vary substantially over time or between locations; see full results in Additional file 1: Fig. S5), as evidenced by the narrow confidence intervals (blue and green shaded areas in Fig. 3). An intensity rate of 0.2 was generally effective, although its efficacy did not exceed 51% and was associated with wider confidence intervals. Regardless of intensity, the intervention’s efficacy was lower during the initial months than during the rest of the summer. When performed at an intensity rate of 0.2, efficacy slightly decreased from September onwards.

The second most efficient intervention strategy was larvicide treatment (Fig. 3iii). This intervention showed no efficacy until June, but from June onwards, the efficacy increased, reaching values comparable to those of mosquito breeding site reduction (with maximum efficacies of 47.7%, 87.1% and 95.7% for intervention intensity rates of 0.2, 0.5 and 0.8, respectively). Additionally, the efficacy of larvicide treatment at intensity rates of 0.5 and 0.8 was consistent across years and areas.

Adulticide treatment (Fig. 3ii), although able to reduce the number of circulating adult *Cx. pipiens* mosquitoes (with maximum efficacy of 32.8%, 71.2% and 84.3% intervention intensity rates of 0.2, 0.5 and 0.8, respectively), was less effective but constant across years and areas. The elimination of overwintering mosquitoes (Fig. 3i) was less effective and decreased over the summer. Maximum efficacy was achieved in May/June, reaching 12.9%, 35.1% and 63% for intensity rates of 0.2, 0.5 and 0.8, respectively. However, by late September, efficacy decreased to minimum values of −27.3%, −14.6% and 58.2%, respectively. This intervention exhibited greater variability across years and areas, as indicated by the wider confidence intervals.

All interventions on birds (active removal of both competent and non-competent birds or reduction of their breeding sites; Fig. 3v–x) showed no efficacy until June. After June, efficacy decreased for all interventions, indicating an increase in the number of infectious mosquitoes. The active removal of competent birds and all birds (Fig. 3vi, vii) and the reduction in breeding sites for competent birds (Fig. 3ix) had wider confidence intervals, indicating greater variability in efficacy across years and areas.

## Discussion

Our study presents a framework for evaluating the seasonal efficacy of ten intervention strategies aimed at reducing the number of WNV-infected mosquitoes. We found that mosquito-targeted interventions are consistently more effective than targeting hosts (birds), which, in contrast, may inadvertently increase outbreak sizes in mosquitoes. Among mosquito interventions, daily efforts focused on immature life stages – such as reducing the number of breeding sites and eliminating eggs or larvae – proved to be the most effective. While reducing adult mosquito populations can lower infection rates, its efficacy was slightly lower than that of interventions targeting non-adult mosquitoes, whereas reducing the number of overwintering mosquitoes was the least effective strategy. Moreover, our results indicate that the efficacy of an intervention plan is influenced by its intensity rate; however, for all tested interventions, the efficacy varied over time during the simulation period, and some interventions and intensity rates were more sensitive to variations across years and areas, indicating greater variability in their simulated efficacy (Additional file 1).

In line with the literature on vector-borne diseases, we confirmed that the most effective intervention strategy is vector control [17, 39]. Although the results presented are not novel per se – as vector control remains the cornerstone of managing most vector-borne infections – our findings underscore the value of tailored studies in enhancing the efficiency of interventions aimed at reducing human infection risk. Data collection remains a critical component for informing targeted strategies, as reflected by the wide confidence intervals in our simulations. However, when robust data collection is coupled with modelling efforts, a more site-specific and effective approach to controlling vector-borne infections becomes feasible. Furthermore, our results demonstrate that increases in intervention efficacy are not linearly related to the intensity of application and that efficacy varies over the course of the season. This highlights the importance of complementing established practices with locally tailored analyses that account for the specific ecological and climatic conditions of the area – especially in the context of ongoing climate and land-use changes.

In particular, the elimination of eggs and larvae – which can be performed through measures such as reducing mosquito breeding sites or applying insecticides and treatments targeting immature life stages – can significantly diminish the size of WNV outbreaks. We also found that the efficacy of the tested treatments for eliminating infectious mosquitoes increased with increasing application intensity, although this relationship was not linear. For instance, our modelling indicates no substantial difference in the elimination of non-adult mosquitoes at application rates of 0.5 and 0.8. This suggests that achieving an application rate of 0.8 may not be necessary and, in light of the environmental impact of insecticide usage, underscores the importance of quantitative analyses to optimise resource allocation and balance treatment efficacy. Our framework also shows that both the elimination of mosquito breeding sites and the active killing of larvae and eggs are efficient strategies. Both chemical and mechanical methods are currently available; however, due to the toxicity of the former, the latter may be considered a more suitable option. However, mechanical reduction of mosquito breeding sites is associated with high maintenance costs. It may prove impractical, as it necessitates the reclamation of wetlands and the elimination of stagnant water, both of which depend on environmental and climatic conditions. Additionally, breeding sites may be situated on private property (such as flowerpots), requiring the involvement of private landowners in intervention planning. Alternative methods, such as bacterial larvicides including *Bacillus thuringiensis* subsp. *israelensis* (Bs) (Bti) and *B. sphaericus*, offer high specificity against Diptera larvae (e.g. mosquitoes), minimising environmental impact while effectively reducing adult mosquito emergence. Although knowledge gaps remain regarding their interactions with abiotic factors (e.g. temperature) and biotic variables (e.g. ecological differences), these methods show significant promise [40]. Therefore, our results suggest that new tools to reduce the number of eggs and larvae, or a dedicated effort to achieve this goal, may play a key role in controlling mosquito-borne infections. Our observations indicate that increasing the mortality of mosquito eggs and larvae, for instance, by using chemical larvicides, leads to a reduction in the number of infectious mosquitoes comparable to that achieved by reducing breeding sites. The traditionally used products include growth regulators or synthetic neurotoxins [41–44]. The former are less toxic to other insect species, but they are also effective only during the mosquito moulting period and thus have a limited effect over time. The latter are continuously efficient but tend to persist in the environment and negatively affect the development of invertebrates, amphibians, reptiles and birds. Our analysis suggests that even low-intensity treatments can effectively reduce the number of infectious mosquitoes, suggesting that mild application of chemical products or the use of growth regulators might be a good option to reduce WNV circulation with less harmful effects on the environment. However, our study assumes constant application of the intervention over time and a uniform effect of the treatment on larvae and eggs (without distinguishing moulting states); therefore, further analyses are needed to confirm the real efficacy of growth regulators over time. Biological control, such as the introduction of natural larval predators (e.g. fishes or amphibians), has shown promising results in controlling vector abundance [44–47]. However, further investigations are needed to identify suitable species specific to each area. New mathematical models could be developed to test the efficacy of larvicidal control through natural predation. Still, each ecological niche is unique and requires the development of an ad hoc model to study its dynamics.

Our findings indicate that insecticides targeting adult mosquitoes can also reduce the number of WNV-infected mosquitoes, although they are less effective than treatments against larvae and eggs. Given that chemical insecticides for adult mosquitoes belong to the same class as those used for larvae and eggs and thus have similar toxic effects, they are less suitable as long-term intervention strategies. Additionally, in recent years, there have been a growing number of reports of insecticide resistance in mosquitoes [48–51], underscoring the urgent need to identify and consider alternative intervention strategies.

Eliminating overwintering mosquitoes, for example, by treating areas during winter, appears less effective than other vector-targeted intervention strategies, emphasising the importance of sustained treatments throughout the summer. Given the rapid pace of the mosquito life cycle, under favourable climatic conditions, it takes only a few weeks for a population of a few dozen mosquitoes to expand into thousands.

The controversial effects of reducing the number of birds (both competent and non-competent, either through active removal or removal of breeding sites) that we observed align with previous studies [17, 28, 39] and are also attributable to specific modelling assumptions. In particular, counterintuitive results, also reported in other modelling studies [28], may be explained by increased biting pressure on the remaining birds when host density is reduced under the assumption of frequency-dependent transmission. If frequency dependence is considered a biologically plausible mechanism for WNV transmission between birds and mosquitoes, then reducing bird density could unintentionally amplify transmission dynamics. Additionally, we can interpret these results in light of the fact that the basic reproductive number (*R*₀) of WNV derived from our model is proportional to the vector-to-host ratio (i.e. number of vectors divided by the number of hosts [27, 35]). Therefore, if the number of hosts decreases while the number of vectors remains constant, the ratio increases, leading to a corresponding increase in *R*₀.

Interestingly, interventions targeting bird hosts also displayed the greatest variability in outcomes across years and clusters, as indicated by the wider confidence intervals in Fig. 3 and Fig. S5, particularly towards the end of the transmission season. This suggests that the effectiveness of the strategy may be highly dependent on year- and location-specific conditions, particularly temperature, the intensity of viral circulation at the beginning of the season and mosquito abundance, which were the key variables allowed to vary across simulation years and areas in our framework. In particular, the removal of competent birds, the removal of bird breeding sites and the removal of competent bird breeding sites had beneficial effects for some years and areas. This variability is consistent with the previously discussed mechanisms: Reducing bird abundance increases mosquito biting pressure on the remaining individuals, which may in turn accelerate the depletion of susceptible hosts and drive the system towards saturation, ultimately leading to a decline in transmission. This outcome is particularly relevant, as it highlights the importance of carefully considering local and temporal ecological conditions – such as mosquito abundance and baseline WNV dynamics – when planning bird-targeted interventions. In addition, given the relevance of the assumption of frequency dependence in modelling outcomes [28], the biting rate and mosquito feeding preference have emerged as critical factors for assessing the efficacy of interventions for birds, as they affect the frequency of bites to birds. Given that the biting rate of mosquitoes plays a key role in WNV dynamics [27], the central role of susceptible birds in sustaining WNV transmission, the availability of competent hosts and the extent to which mosquitoes selectively bite birds [52] can substantially affect both the progression of the outbreak and the effectiveness of control strategies. As such, assumptions regarding mosquito feeding behaviour should be carefully evaluated when bird-focused interventions are designed, as they may alter the expected outcomes in complex and non-linear ways. In our study, we considered only a limited range of biting rates; however, exploring a broader spectrum of mosquito feeding behaviours is crucial, as variations in these parameters could lead to substantially different and potentially non-linear outcomes. There are still significant knowledge gaps regarding vector‒host interactions [27], the composition of avian communities and epidemiological and demographic data on bird species, making accurate prediction of the actual local impact of reducing the number of competent birds difficult. Future work should therefore focus on better characterising these aspects to improve the reliability of bird-targeted control measures.

Previous studies [53] have shown that the assumption that the basic reproduction number (*R*₀; defined as the average number of secondary infections produced by a single infectious individual in a completely susceptible population) is less than one guarantees that the extinction of WNV is not always reliable, as disease outbreaks can occur even when *R*₀ < 1 due to local conditions [28]. In our study, we therefore predicted the daily number of infectious mosquitoes as a proxy for human infection risk instead of relying solely on *R*₀. Our findings show that intervention efficacy varies over time, even with constant application intensity. For instance, eliminating overwintering mosquitoes is effective only until August, while removing adults and larvae effectively reduces the number of infectious mosquitoes by the end of summer. Other interventions have short-term effects. Given that human cases increase in late summer and that the risk of infection rises due to a potential shift in mosquito feeding preferences towards humans [54], our results highlight the importance of analysing intervention efficacy over time. Notably, temperature significantly affects mosquito developmental rates [36, 55, 56], the probability of WNV transmission from birds to mosquitoes per infectious bite [57] and the extrinsic incubation period [58]. As a result, temperature exerts a strong influence on the basic reproduction number (*R*₀) of WNV [27, 59, 60]. This underscores the importance of detailed data collection to improve our understanding and prediction of disease spread, as well as the need for studies assessing the efficacy of intervention strategies tailored to specific ecological and climatic contexts. Moreover, in the face of ongoing climate change, further investigations will be essential to enhance our capacity to mitigate WNV transmission and reduce the associated risk to human health.

Moreover, we emphasised that different intervention choices may lead to varying outcomes across years and areas, with higher application rates generally providing more consistent efficacy. In contrast, lower application rates are more dependent on specific system conditions and require a more thorough evaluation, suggesting that more conservative choices may be preferable.

We must acknowledge that our results assume that intervention strategies are applied consistently throughout the summer season, which simplifies the complexity of the process. Therefore, further studies are needed to explore the effects of irregular treatments or varying application intensities throughout the season. Furthermore, a comprehensive assessment of intervention efficacy strategies would require site-specific analysis, as local conditions – such as the size and nature of breeding sites, potential negative feedback on species that naturally regulate mosquito populations and other ecological characteristics of the treated areas – can significantly influence outcomes. Nonetheless, we believe that a general modelling framework such as the one proposed here, although theoretical, can serve as a valuable baseline and a powerful tool to support and inform such targeted analyses.

Additionally, various mosquito species are recognised as suitable vectors for WNV, and multiple bird species have tested positive for the virus. This suggests that the composition of the vector or the avian community may influence the number of mosquitoes and birds that are infected [13, 61–64]. Our model, which accounts for only one vector (*Cx. pipiens*) and one host species, overlooks the interactions among species that could positively or negatively impact the efficacy of intervention strategies.

Further developments of the present study are possible, particularly in light of ongoing climate and environmental changes, which are expected to affect vector abundance, distribution and vector–host community composition, ultimately influencing WNV dynamics and the effectiveness of intervention strategies. In particular, the potential effects of warmer winters on mosquito hibernation and their possible impact on the early-season presence of WNV-infected vectors deserve further investigation. Although this aspect is not currently explored, it represents a promising direction for future research, especially if robust empirical estimates become available to inform model calibration under altered climatic conditions. The modelling framework proposed here could be extended to incorporate a range of climate change scenarios into simulation analyses.

## Conclusions

In conclusion, our work reinforces existing evidence and provides additional insights into the control of mosquito-borne infections, both at a global scale and in local contexts. These findings support the insight that mosquito control is the primary intervention strategy for mitigating WNV outbreaks on a global scale [17, 28] and offer region-specific insights for Lombardy and other areas with similar climatic conditions. Nevertheless, a careful assessment of the timing and intensity of the intervention is fundamental to maximise its efficacy at reducing human infection risk. Indeed, even though the effect of an intervention depends on the intensity applied, the obtained reduction in risk does not increase linearly with the effort applied. Moreover, the efficacy of interventions is not constant over time. For these reasons, mathematical modelling can be an effective tool for evaluating intervention strategies, allowing the investigation of the effects of the interventions over time and hypothetical scenarios, substantially saving time and resources. It is nevertheless important to note that this work does not aim to provide an exhaustive assessment of all possible intervention strategies but rather to apply an established modelling framework to the specific context of the Lombardy region. In doing so, it demonstrates the promise of this approach to inform vector control decisions and underscores the need for a continuous feedback loop between field data collection and modelling [65]. Such an iterative process is crucial both to identify and address knowledge gaps and to establish a virtuous cycle [65] where modelling informs empirical investigations and strategic planning, and empirical data, in turn, enhance model accuracy and relevance.

## Supplementary Information

Additional file 1.

Additional file 2: Recorded average entomological captures for each year and area.

Additional file 3: Total number of analysed mosquito pools for each year and cluster.

Additional file 4: Total number of WNV-positive pools for each year and cluster.

## Data Availability

All equations are provided in detail in Additional File 1 to ensure the reproducibility of the research, and all data are available in Additional Files 2, 3 and 4.
