# Supplementary material for: Are we doing our best to contain the spread of West Nile virus? Evaluating intervention efficacy through mathematical modelling
Source: Parasit Vectors. 2025 Dec 5;18:499. doi: 10.1186/s13071-025-07128-9 (PMC12681172; doi:10.1186/s13071-025-07128-9)
Supplement: Supplementary file 1 — Additional file 1. [file 13071_2025_7128_MOESM1_ESM.pdf]

Supplementary information for: 'Are we doing our best to contain the spread of West Nile virus? Evaluating intervention efficacy through mathematical modelling'

Supplementary methods

Text S1: *Entomological-model*

We estimated the daily mosquito abundance following the system of differential equations here reported:

System 1:

$$\begin{cases} E'(t) = n_E \cdot d_A \cdot A(t) - (\mu_E + \tau_E) \cdot E(t) \\ L'(t) = \tau_E \cdot E(t) - (\tau_L + \mu_L \cdot (1 + \frac{L(t)}{K})) \cdot L(t) \\ P'(t) = \tau_L \cdot L(t) - (\tau_P + \mu_P) \cdot P(t) \\ A'(t) = (1 - \delta) \cdot 0.5 \cdot P(t) - \mu_A \cdot A(t) \end{cases}$$

In the proposed system E, L, P and A respectively represent eggs, larvae, pupae and adult non-diapausing female mosquitoes. Death rates (μ_E, μ_L, μ_P and μ_A) and developmental rates (τ_E, τ_L, and τ_P) specific of each age-class are all considered temperature dependent, in accordance with the work proposed by Marini (1).

Here n_E represents the number of eggs laid in one oviposition, whereas δ represents the fraction of mosquitoes that undergo the diapause. Two different density dependant scaling factors driving the carrying capacity for the larval stage (K) were included, one for the early part of the season (up to June 30) and a different one for the late part of the season (from June 30) due to a possible change in *Cx. pipiens* breeding sites availability due to competition for resources with *Ae. Albopictus* (2)Considering only adult female mosquitoes, the term 0.5 in the equation for adults accounts for the sex ratio. Since traps capture host seeking mosquitoes, only a fraction d_A of adult mosquitoes is considered to lay eggs.

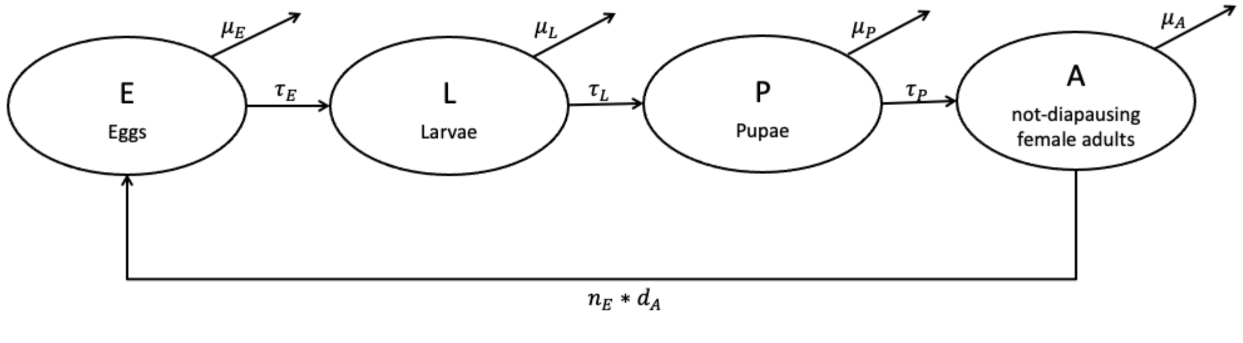

Figure S1: Entomological-model scheme. Model flow chart for mosquito dynamics. Compartments (circles) represent four developmental stages of mosquitoes: eggs (E), larvae (L), pupae (P) and not-diapausing female adults (A).

Text S2: ***Epidemiological-model***

According with the scheme reported in Figure S2 we simulated WNV spread into Lombardy region through the following system of differential equations:

System 2:

$$
\begin{cases}
M'_S(t) = \omega(t) - \left(b \cdot p \cdot p_{BM} \cdot \dfrac{B_{Ia}(t) + B_{Ij}(t)}{B_T(t) + N_T(t)} + \mu_M\right) \cdot M_s(t) \\
M'_E(t) = b \cdot p \cdot p_{BM} \cdot \dfrac{B_{Ia}(t) + B_{Ij}(t)}{B_T(t) + N_T(t)} \cdot M_s(t) - (\theta_M + \mu_M) \cdot M_E(t) \\
M'_I(t) = \theta_M \cdot M_E(t) - \mu_M \cdot M_I(t) \\
B'_{Sa}(t) = -\left(b \cdot p_{MB} \cdot \dfrac{M_I(t)}{B_T(t) + N_T(t)} + \mu_B\right) \cdot B_{Sa}(t) \\
B'_{Ea}(t) = b \cdot p_{MB} \cdot \dfrac{M_I(t)}{B_T(t) + N_T(t)} \cdot B_{Sa}(t) - (\mu_B + \theta_B) \cdot B_{Ea}(t) \\
B'_{Ia}(t) = \theta_B \cdot B_{Ea}(t) - (\mu_B + \sigma_B) \cdot B_{Ia}(t) \\
B'_{\square\square a}(t) = \sigma_B \cdot B_{Ia}(t) - \mu_B \cdot B_{Ra}(t) \\
B'_{Sj}(t) = \left(\gamma - r \cdot \dfrac{B_j(t)}{K}\right) \cdot B_a(t) - \left(b \cdot p_{MB} \cdot \dfrac{M_I(t)}{B_T(t) + N_T(t)} + \left(\mu_{Bj} + r \cdot \dfrac{B_j(t)}{K}\right)\right) \cdot B_{Sj}(t) \\
B'_{Ej}(t) = b \cdot p_{MB} \cdot \dfrac{M_I(t)}{B_T(t) + N_T(t)} \cdot B_{Sj}(t) - \left(\left(\mu_{Bj} + r \cdot \dfrac{B_j(t)}{K}\right) + \theta_B\right) \cdot B_{Ej}(t) \\
B'_{Ij}(t) = \theta_B \cdot B_{Ej}(t) - \left(\left(\mu_{Bj} + r \cdot \dfrac{B_j(t)}{K}\right) + \sigma_B\right) \cdot B_{Ij}(t) \\
B'_{Rj}(t) = \sigma_B \cdot B_{Ij} - \left(\mu_{Bj} + r \cdot \dfrac{B_j}{K}\right) \cdot B_{Rj} \\
N'_a(t) = -\mu_{Na} \cdot N_a(t) \\
N'_j(t) = \left(\gamma_N - r \cdot \dfrac{N_j(t)}{K}\right) \cdot N_a(t) - \left(\mu_{Nj} + r \cdot \dfrac{N_j(t)}{K}\right) \cdot N_j(t)
\end{cases}
$$

In the proposed system M_S, M_E and M_I respectively represent the susceptible, exposed and infectious mosquito population, whereas B_{Sa}, B_{Ea}, B_{Ia} and B_{Ra} susceptible, exposed, infectious and recovered competent adult birds and B_{Sj}, B_{Ej}, B_{Ij} and B_{Rj} susceptible, exposed infectious and recovered competent juvenile birds. B_T represents the total competent avian community, B_a are the adults, and thus sexually mature competent birds, whereas B_j are the young, and thus not yet sexually mature, competent birds. Analogously, N_T are the total non-competent birds, with N_a representing the adults, and N_j the young.

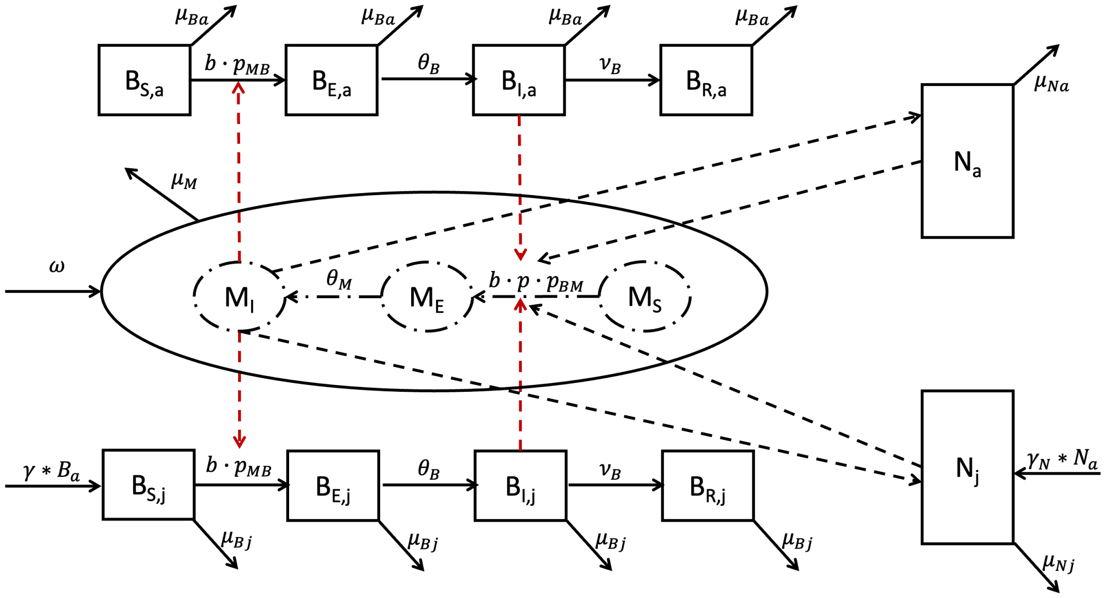

Figure S2: Model scheme. Model flow chart for WNV transmission in birds (squares) and mosquitoes (circles) in an average trapped area. Compartments: $B_{S,a}$, $B_{E,a}$, $B_{I,a}$ and ($B_{S,j}$, $B_{E,j}$, $B_{I,j}$ and $B_{R,j}$): adult (juvenile) susceptible, exposed, infectious and immune birds; Ms, Me, Mi: susceptible, exposed and infectious mosquitoes; N_a and N_j: adult and juvenile non-competent birds. Solid arrows represent epidemiological processes whereas dashed arrows represent the influence of a compartment on a process.

Model parameters

Table S1: Model parameters for the base entomological model

Parameter	Biological meaning	Value	Source
τ_E	Developmental rate for eggs (day^{-1})	$2.2 \cdot 10^{-3} T^{1.77}$	(3)
τ_L	Developmental rate for larvae (day^{-1})	$\frac{1}{93.24-6.83T+0.13T^2}$	(4,5)
τ_P	Developmental rate for pupae (day^{-1})	$\frac{1}{20.17e^{-0.096T}}$	(4,5)
d_A	Gonotrophic cycle length	$\frac{1}{0.122(log_{10}(T-9))^{1.76}}$	(3)
μ_E	Death rate for eggs (day^{-1})	$0.095e^{\left(\frac{T-22.88}{7}\right)^2}$	(3)
μ_L	Death rate for larvae (day^{-1})	$2.7 \cdot 10^{-2} + 3 \cdot 10^{-9} e^{0.64T}$	(4,5)
μ_P	Death rate for pupae (day^{-1})	$2 \cdot 10^{-5}(T - 20.08)^4 + 0.08$	(4)

$\boldsymbol{\mu_A}$	Death rate for adults (day^{-1})	$\frac{4.61}{151.6-4.57T}$	(4)
$\boldsymbol{d_P}$	Fraction of emerging diapausing adults	$\frac{1+e^{\frac{829.86-1}{30.07}}}{1+0.5e^{\frac{829.86-1}{30.07}}}-1$	(4)
$\boldsymbol{n_E}$	Number of eggs laid in one oviposition	200	(3,4)
$\boldsymbol{d_P}$	Daily capture rate	0.108	(6)
$\boldsymbol{r}$	Daily average mosquito flight range(meters)	500	(7,8)

In the table T represents the daily mean temperature

Table S2: Model parameters for the base epidemiological model

Parameter	Biological meaning	Value	Source
μ_M	Mosquito death rate (day^{-1})	$\frac{4.61}{151.6-4.75\cdot T}$	(3)
p_{BM}	Probability of WNV transmission from bird to mosquito per infectious bite	$\frac{e^{(-10.917+0.365\cdot T)}}{1+e^{(-10.917+0.365\cdot T)}}$	(9)
p_{MB}	Probability of WNV transmission from mosquito to bird per infectious bite	0.94	(10)
θ_M	Extrinsic incubation period (day^{-1})	$\frac{1}{0.0092T-0.132}$	(11)
θ_B	Intrinsic incubation period (day^{-1})	0.5	(10)
$\gamma_B(t)$	Fertility rate for competent birds (magpies) at day t (day^{-1})	0.2 (t ≤ July 20)<br>0 (t > July 20)	(12)
$\gamma(t)$	Fertility rate for non-competent birds at day t (day^{-1})	0.2 (t ≤ July 20)<br>0 (t > July 20)	*
μ_{Ba}	Death rate of adult competent birds (magpies, day^{-1})	0.001	(12)
μ_{Bj}	Death rate of juvenile competent birds (magpies, day^{-1})	0.003	(12)
μ_{Na}	Death rate of adult non-competent birds (day^{-1})	0.001	*
μ_{Nj}	Death rate of juvenile non-competent birds (day^{-1})	0.003	*

In the table T represents the daily mean temperature

*expert opinion

Table S3: Prior distribution of unknown parameter ranges

Parameter	Biological meaning	Value 95%CI
$\boldsymbol{B_0}$	Initial number of birds in the area surrounding the trap	50-70
$\boldsymbol{a_i}$	Proportion of competent birds	0.2-0.8
$\boldsymbol{b}$	Proportion of mosquito bites on birds	0.5-0.9
$\boldsymbol{M_{I0}}$	Prevalence of infectious mosquitoes at the beginning of the season (April)	0-0.1%
$\boldsymbol{B_{R0}}$	Initial number of immune birds at the beginning of the season (April)*	0-100%
$\boldsymbol{\sigma_B}$	Recovery rate (day^{-1}), i.e. the inverse of the mean duration of the infection	0.1-0.5

* Between 0–100% in 2016, while for 2017 and 2018, it was adjusted to ±20% of the values recovered from the previous year.

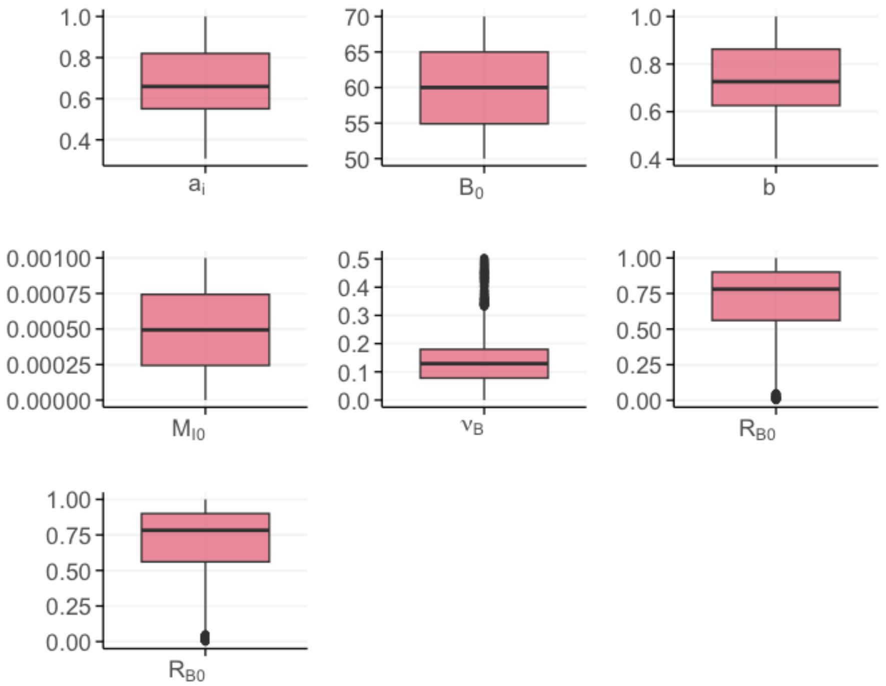

Figure S3: Posterior distribution of unknown parameter ranges. Mean and 95%CI of the 100 estimates for the unknown parameters.

Supplementary results

Text S3: *Fit of the base model*

The proposed model showed to efficiently describe WNV dynamics in Lombardy region, including the 98% of the total (considering all years and clusters) observations within the 95% Confidence Interval predictions of the model (figure D). Confidence intervals of the predicted infected mosquitoes are wide, reflecting the variability in parameter values used for simulations (Supplementary materials).

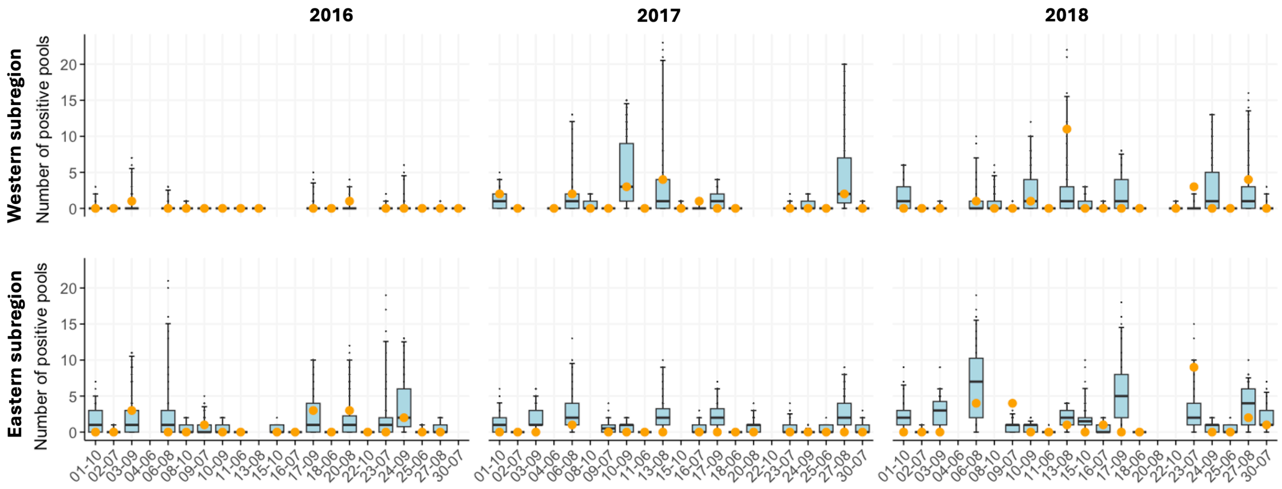

Figure S4: Model prediction fit. Predicted number of WNV-positive pools for the three years (2016, 2017 and 2018, panels from left to right) and the two subregions of Lombardy where WNV circulated in the triennium of analysis (top-bottom panels). Orange points: observed weekly number of WNV-positive pools; lightblue boxplots (median, 2.5 and 97.5% quantiles): the predicted distribution of positive pools per week.

Text S4: ***Intervention strategies inclusion***

Intervention strategies on the vector population were included in model equations by adjusting *System 1* as follows:

System 3:

$$\begin{cases} E'(t) = n_E \cdot d_A \cdot A(t) - (\mu_E + \tau_E) \cdot E(t) - i_2 \cdot E(t) \\ L'(t) = \tau_E \cdot E(t) - \left(\tau_L + \mu_L \cdot \left(1 + \frac{L}{K \cdot i_4}\right)\right) \cdot L(t) - i_2 \cdot L(t) \\ P' = \tau_L \cdot L(t) - (\tau_P + \mu_P) \cdot P(t) \\ A'(t) = (1 - \delta) \cdot 0.5 \cdot L(t) - \mu_A \cdot A(t) - i_3 \cdot A(t) \end{cases}$$

Where i_2, i_3 and i_4, respectively represent the intensity rate of the larvicide treatment, the adulticide treatment and of the reduction of the mosquito breeding sites (strategies *b-d*).

Intervention strategies on the host population were included in model equations by adjusting *System 2* as follows:

System 4:

$$\begin{cases} M'_S(t) = \omega(t) - (b \cdot p \cdot p_{BM} \cdot \frac{B_{Ia}(t) + B_{Ij}(t)}{B_T(t)} + \mu_M) \cdot M_s(t) \\ M'_E(t) = b \cdot p \cdot p_{BM} \cdot \frac{B_{Ia}(t) + B_{Ij}(t)}{B_T(t)} \cdot M_s(t) - (\theta_M + \mu_M) \cdot M_E(t) \\ M'_I(t) = \theta_M \cdot M_E(t) - \mu_M \cdot M_I(t) \\ B'_{Sa}(t) = -\left(b \cdot p_{MB} \cdot \frac{M_I(t)}{B_T(t)} + \mu_{Ba}\right) \cdot B_{Sa}(t) - i_5 \cdot B_{Sa}(t) \\ B'_{Ea}(t) = b \cdot p_{MB} \cdot \frac{M_I(t)}{B_T(t)} \cdot B_{Sa}(t) - (\mu_B + \theta_B) \cdot B_{Ea}(t) - i_5 B_{Ea}(t) \\ B'_{Ia}(t) = \theta_B \cdot B_{Ea}(t) - (\mu_B + \sigma_B) \cdot B_{Ia}(t) - i_5 \cdot B_{Ia}(t) \\ B'_{Ra}(t) = \sigma_B \cdot B_{Ia}(t) - \mu_B \cdot B_{Ra}(t) - i_5 \cdot B_{Ra}(t) \\ B'_{Sj}(t) = \left(\gamma_B(t) - r \cdot \frac{B_j}{K \cdot i_6}\right) \cdot B_a(t) - (b \cdot p_{MB} \cdot \frac{M_I(t)}{B_T(t)} + (\mu_{Bj} + r \cdot \frac{B_j(t)}{K \cdot i_6})) \cdot B_{Sj}(t) - i_5 \cdot B_{Sj}(t) \\ B'_{Ej}(t) = b \cdot p_{MB} \cdot \frac{M_I(t)}{B_T(t)} \cdot B_{Sj}(t) - ((\mu_{Bj} + r \cdot \frac{B_j(t)}{K \cdot i_6}) + \theta_B) \cdot B_{Ej}(t) - i_5 \cdot B_{Ej}(t) \\ B'_{Ij}(t) = \theta_B \cdot B_{Ej}(t) - ((\mu_{Bj} + r \cdot \frac{B_j(t)}{K \cdot i_6}) + \sigma_B) \cdot B_{Ij}(t) - i_5 \cdot B_{Ij}(t) \\ B'_{Rj}(t) = \sigma_B \cdot B_{Ij}(t) - (\mu_{Bj} + r \cdot \frac{B_j(t)}{K \cdot i_6}) \cdot B_{Rj}(t) - i_5 \cdot B_{Rj}(t) \\ N'_a(t) = -\mu_N \cdot N_a(t) - i_7 \cdot N_a(t) \\ N'_j(t) = \left(\gamma_N(t) - r \cdot \frac{N_j(t)}{K_N \cdot i_8}\right) N_a(t) - (\mu_{Nj} + r \cdot \frac{N_j(t)}{K_N \cdot i_8}) \cdot N_j(t) - i_7 \cdot N_j(t) \end{cases}$$

Where i_5 and i_6, represent the intensity rate of the interventions affecting the competent bird population, respectively: the removal of competent birds and the reduction of their breeding sites

(strategies *e,f,h,i*). Analogously, i_7 and i_8, represent the intensity rate of the interventions affecting the non-competent bird population, respectively: the removal of non-competent birds and the reduction of their breeding sites (strategies *e,g,h,j*).

Efficacy of the intervention strategies per year and area

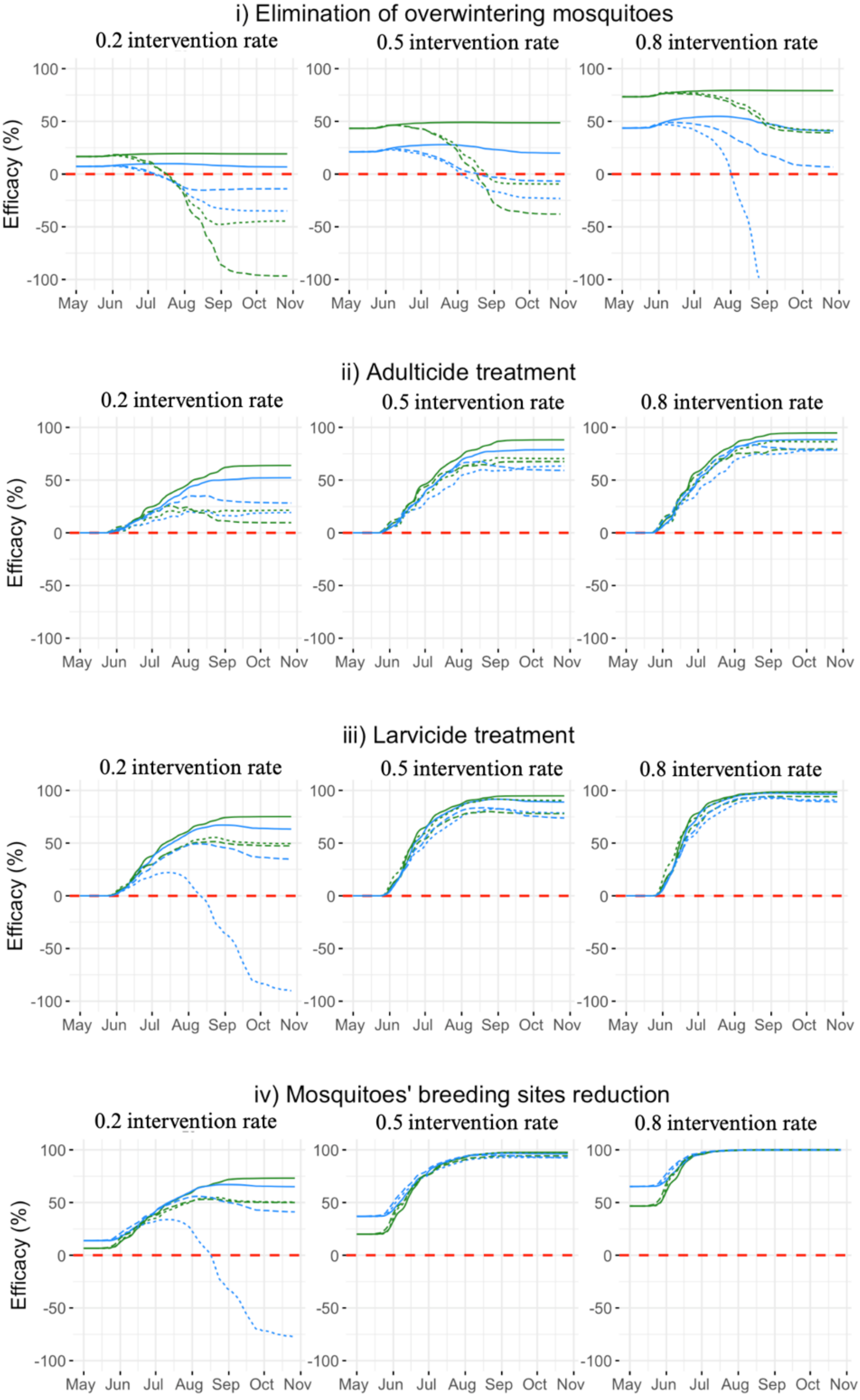

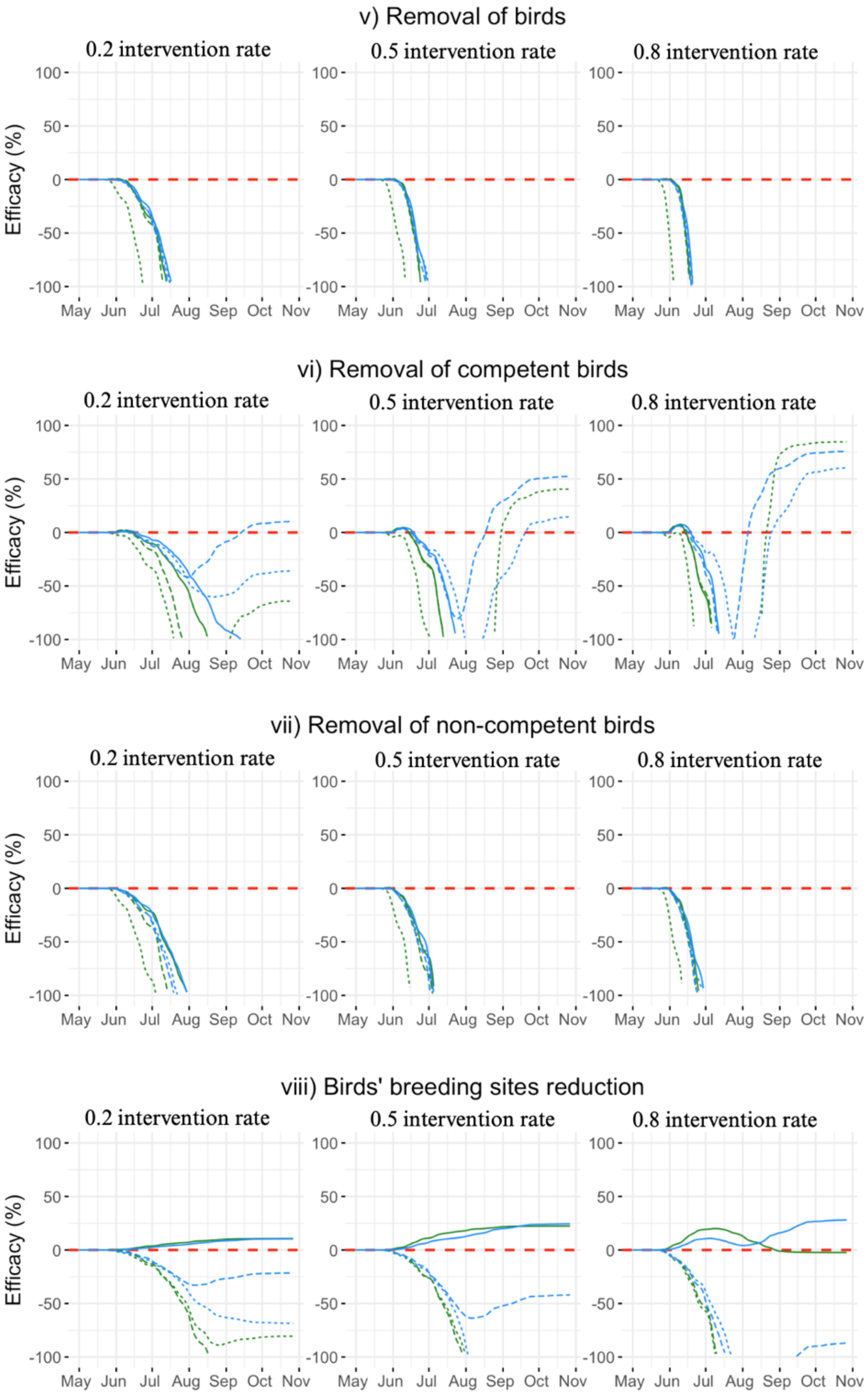

v) Removal of birds
0.2 intervention rate
0.5 intervention rate
0.8 intervention rate
vi) Removal of competent birds
0.2 intervention rate
0.5 intervention rate
0.8 intervention rate
vii) Removal of non-competent birds
0.2 intervention rate
0.5 intervention rate
0.8 intervention rate
viii) Birds' breeding sites reduction
0.2 intervention rate
0.5 intervention rate
0.8 intervention rate
Efficacy (%)
100
50
0
-50
-100
May
Jun
Jul
Aug
Sep
Oct
Nov

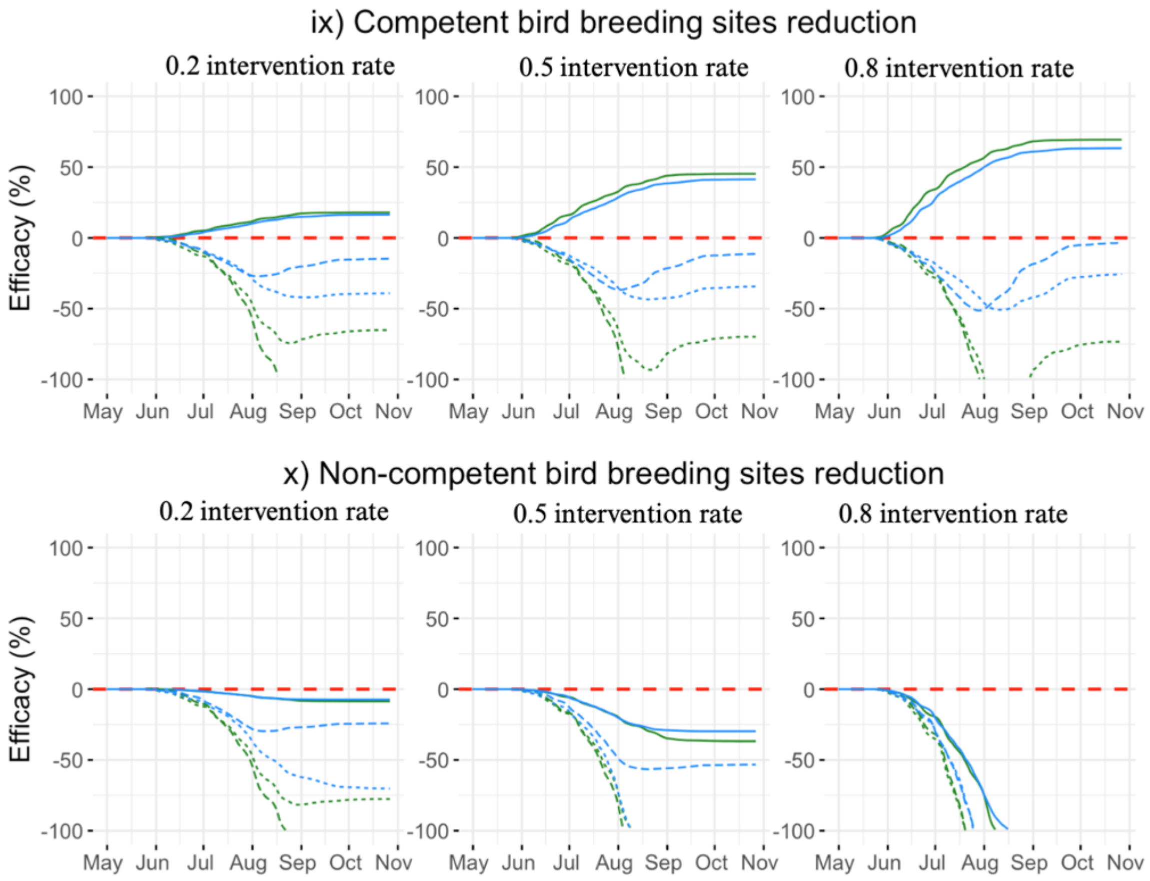

Figure S5: **Efficacy of the intervention strategies over time per cluster and year**. Each row illustrates the average efficacy on the 100 simulations (Ei, on the y-axis) of a specific intervention strategy in reducing the number of infectious mosquitoes over time (x-axis) at three different intervention rates. The colors correspond to the analysis areas (green for the western sub-region and blue for the eastern sub-region), while the line types represent the three simulation years (solid, dotted, and dashed lines for 2016, 2017, and 2018, respectively). The red dashed line indicates the threshold (Ei = 0), distinguishing effective interventions (Ei > 0) from ineffective ones (Ei < 0).

Text S6: ***Detailed Results on the Predicted Number of Infectious Mosquitoes***

The predicted number of infectious mosquitoes in the absence of intervention strategies (Figure S6) remains generally low, particularly before July and after October, with noticeable variability across years and between sub-regions. In 2016, the mean number of infectious mosquitoes is the lowest overall, never exceeding 15. This is especially evident in the western sub-region, where the mean number consistently remains below 2 throughout the season.

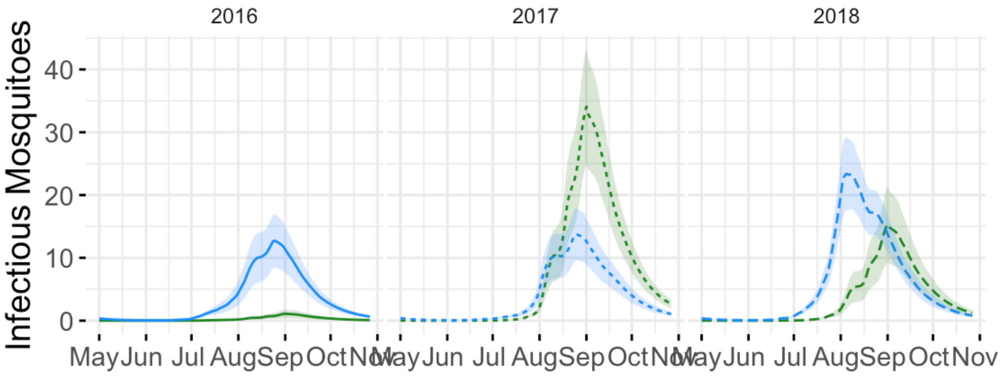

Figure S6. Number of predicted infectious mosquitoes across years and areas. Average daily number of infectious mosquitoes over 100 simulations, shown for each of the three simulation years (three panels) and for the two study areas. Lines represent the mean across simulations, and shaded areas indicate the 95% confidence interval. Colours denote the two sub-regions: green for the western sub-region and blue for the eastern sub-region.

According to the main results, the interventions that most effectively reduce the number of infectious mosquitoes are: the *reduction of mosquito breeding sites (iv)*, the *elimination of eggs and larvae (iii)*, and the *application of adulticide treatments (ii)*. In contrast, the *elimination of overwintering mosquitoes (i)* has little impact on the number of infectious mosquitoes at the seasonal peak. Bird-targeted interventions have only a limited effect when focused on competent species, while targeting all birds or specifically non-competent species can actually increase the number of infectious mosquitoes.

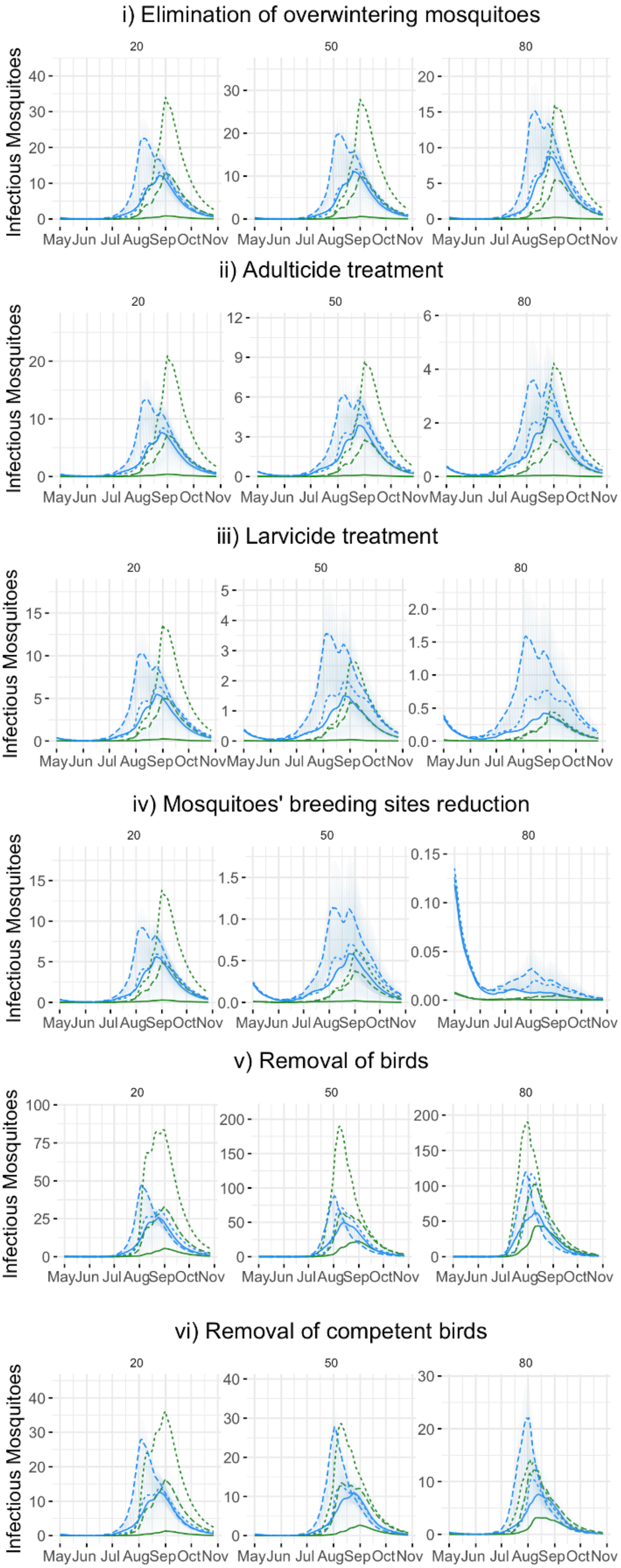
i) Elimination of overwintering mosquitoes
20
50
80
Infectious Mosquitoes
May Jun Jul Aug Sep Oct Nov
ii) Adulticide treatment
20
50
80
Infectious Mosquitoes
May Jun Jul Aug Sep Oct Nov
iii) Larvicide treatment
20
50
80
Infectious Mosquitoes
May Jun Jul Aug Sep Oct Nov
iv) Mosquitoes' breeding sites reduction
20
50
80
Infectious Mosquitoes
May Jun Jul Aug Sep Oct Nov
v) Removal of birds
20
50
80
Infectious Mosquitoes
May Jun Jul Aug Sep Oct Nov
vi) Removal of competent birds
20
50
80
Infectious Mosquitoes
May Jun Jul Aug Sep Oct Nov

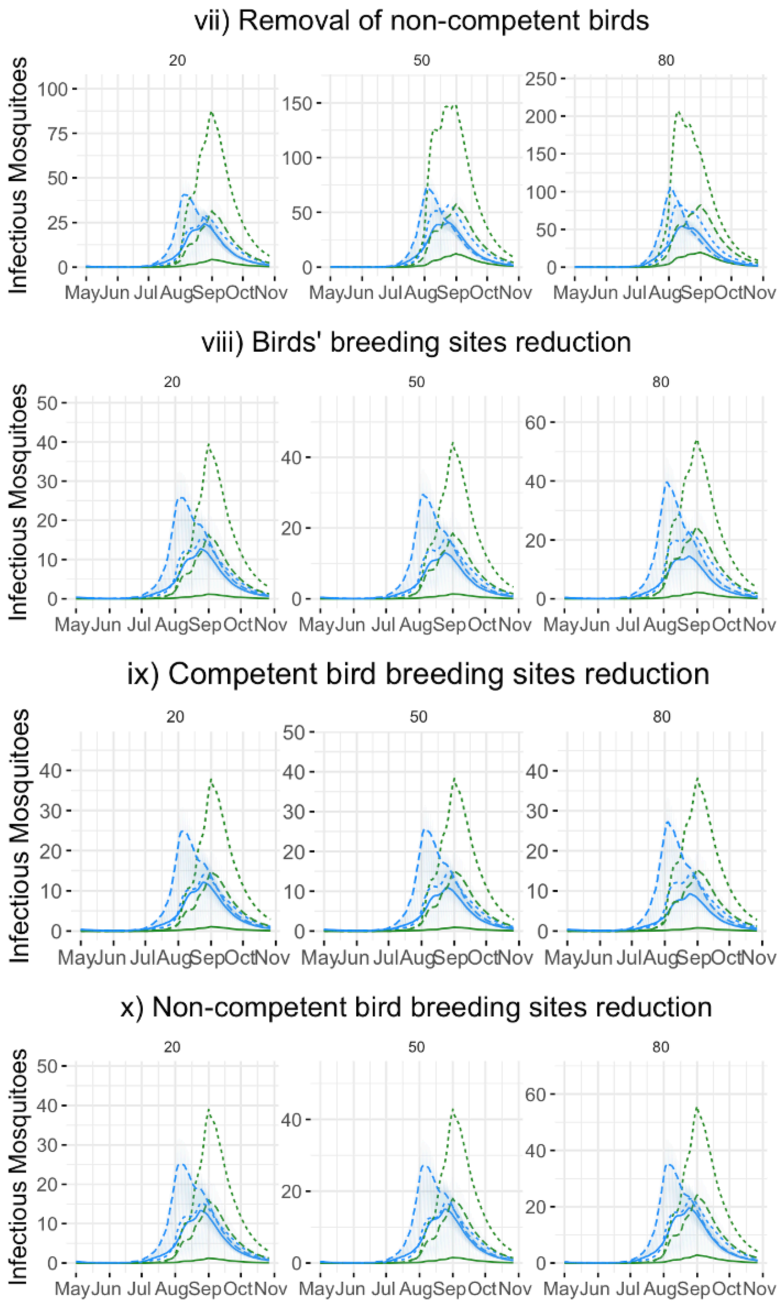

Figure S7. Predicted number of infectious mosquitoes across years and sub-regions under different intervention scenarios. Average daily number of infectious mosquitoes from 100 simulations is shown for each of the three simulated years (solid, dotted, and dashed lines represent 2016, 2017, and 2018, respectively) and for the two study sub-regions (green for the western sub-region and blue for the eastern sub-region). Lines indicate the mean across simulations; shaded areas represent the 95% confidence intervals. Each panel in each row presents results for a different intensity level of the intervention strategy.

To focus on the interventions with the most critical impact and highest variability, we report here the predicted number of infectious mosquitoes from May to November across the three years and two sub-areas of investigation with no intervention and under intervention *(v) Removal of birds*, for each of the 100 parameter sets considered (different colours in Figure S8). We observed that, under

the *no-intervention scenario*, most parameter sets yield similar infection dynamics across years and areas (within a given year), particularly around the peak of the transmission season. However, at the beginning and end of the season, the dynamics tend to diverge more markedly across simulations, years, and areas, with several trajectories approaching near-zero values.

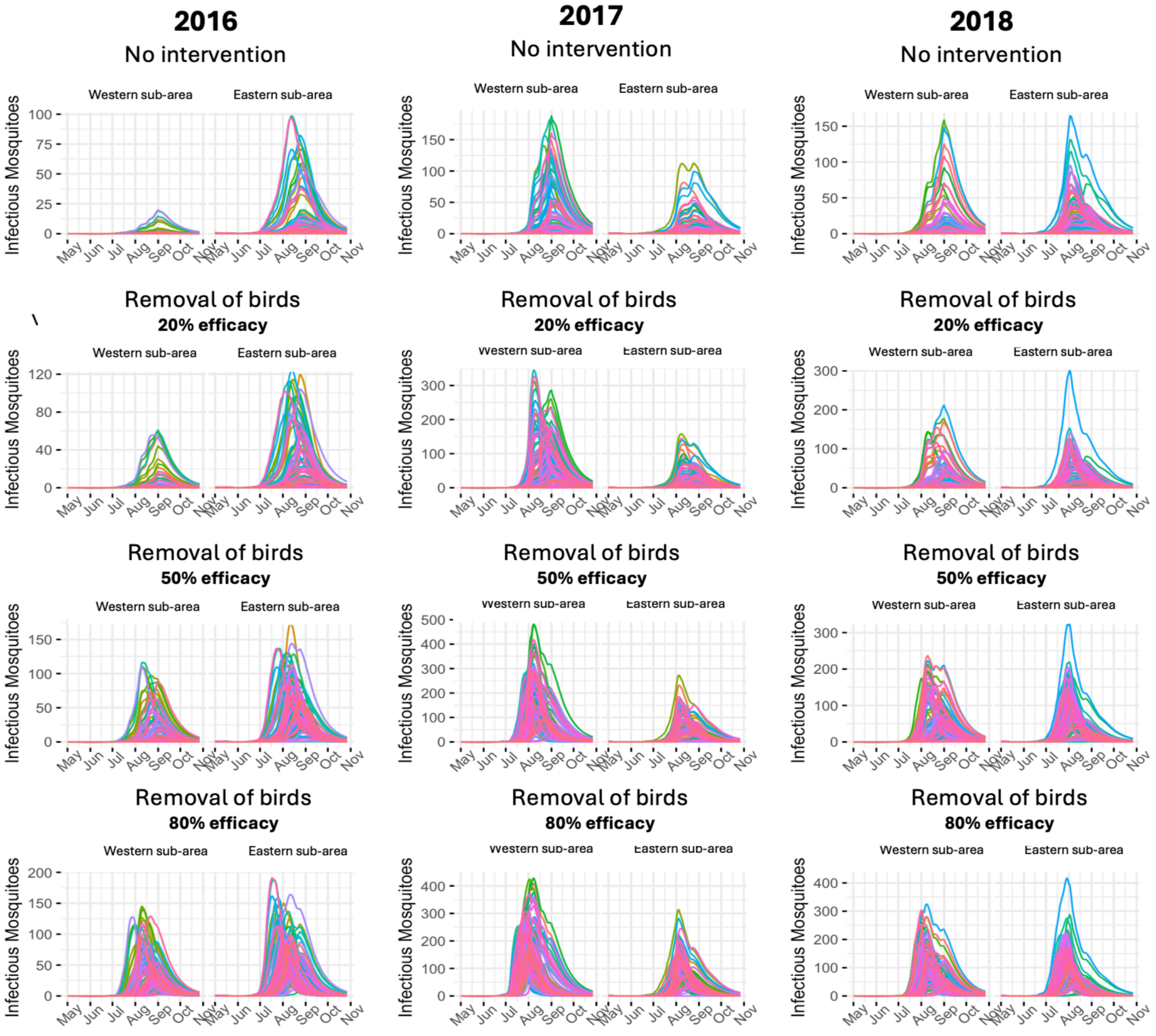

Figure S8. Detail of the predicted number of infectious mosquitoes under intervention v) Removal of birds. Daily number of infectious mosquitoes over 100 simulations (each line represents one simulation), shown for each of the three simulation years (columns) and the two sub-regions (panels). Each row represents a different efficacy of intervention efficacy.

References

1. Marini G, Calzolari M, Angelini P, Bellini R, Bellini S, Bolzoniid L, et al. A quantitative comparison of West Nile virus incidence from 2013 to 2018 in Emilia-Romagna, Italy. 2020;
2. Marini G, Guzzetta G, Baldacchino F, Arnoldi D, Montarsi F, Capelli G, et al. The effect of interspecific competition on the temporal dynamics of Aedes albopictus and Culex pipiens. Parasit Vectors. 2017 Feb 23;10(1):1–9.
3. Ewing DA, Cobbold CA, Purse B V, Nunn MA, White SM. Modelling the effect of temperature on the seasonal population dynamics of temperate mosquitoes. 2016 [cited 2024 Jan 19]; Available from: http://creativecommons.org/licenses/by/4.0/
4. Marini G, Poletti P, Giacobini M, Pugliese A, Merler S, Rosà R. The Role of Climatic and Density Dependent Factors in Shaping Mosquito Population Dynamics: The Case of Culex pipiens in Northwestern Italy. PLoS One [Internet]. 2016 Apr 1 [cited 2024 Dec 31];11(4):e0154018. Available from: https://journals.plos.org/plosone/article?id=10.1371/journal.pone.0154018
5. Loetti V, Schweigmann N, Burroni N. Development rates, larval survivorship and wing length of culex pipiens (Diptera: Culicidae) at constant temperatures. J Nat Hist. 2011;45(35–36):2203–13.
6. Li Y, Su X, Zhou G, Zhang H, Puthiyakunnon S, Shuai S, et al. Comparative evaluation of the efficiency of the BG-Sentinel trap, CDC light trap and Mosquito-oviposition trap for the surveillance of vector mosquitoes. Parasit Vectors. 2016 Aug 12;9(1):1–8.
7. Ciota AT, Drummond CL, Ruby MA, Drobnack J, Ebel GD, Kramer LD. Dispersal of Culex Mosquitoes (Diptera: Culicidae) From a Wastewater Treatment Facility. J Med Entomol. 2012 Jan 1;49(1):35–42.
8. Tsuda Y, Komagata O, Kasai S, Hayashi T, Nihei N, Saito K, et al. A Mark–Release–Recapture Study on Dispersal and Flight Distance of Culex pipiens pallens in an Urban Area of Japan. https://doi.org/102987/57541. 2008 Sep 1;24(3):339–43.
9. Vogels CBF, Fros JJ, Göertz GP, Pijlman GP, Koenraadt CJM. Vector competence of northern European Culex pipiens biotypes and hybrids for West Nile virus is differentially affected by temperature. Parasit Vectors. 2016 Jul 7;9(1):1–7.
10. Del Amo J, Llorente F, Figuerola J, Soriguer RC, Moreno AM, Cordioli P, et al. Experimental infection of house sparrows (Passer domesticus) with West Nile virus isolates of Euro-Mediterranean and North American origins. Vet Res. 2014 Mar 19;45(1):1–9.
11. Reisen WK, Fang Y, Martinez VM. Effects of Temperature on the Transmission of West Nile Virus by Culex tarsalis (Diptera: Culicidae). J Med Entomol. 2006 Mar 1;43(2):309–17.
12. Birkhead T. The Magpies : The Ecology and Behaviour of Black-Billed and Yellow-Billed Magpies. The Magpies : The Ecology and Behaviour of Black-Billed and Yellow-Billed Magpies. Bloomsbury Publishing; 1991.
